# How Does a Shared Decision-Making (SDM) Intervention for Oncologists Affect Participation Style and Preference Matching in Patients with Breast and Colon Cancer?

**DOI:** 10.1007/s13187-016-1146-7

**Published:** 2016-12-13

**Authors:** Christiane Bieber, Jennifer Nicolai, Kathrin Gschwendtner, Nicole Müller, Katrin Reuter, Angela Buchholz, Birgit Kallinowski, Martin Härter, Wolfgang Eich

**Affiliations:** 1 Department of General Internal Medicine and Psychosomatics, Center for Psychosocial Medicine, Heidelberg University Hospital, Thibautstraße 4, 69115 Heidelberg, Germany; 2 Department of Psychology III - Cognition and Individual Differences, University of Mannheim, Mannheim, Germany; 3 Department of Psychiatry and Psychotherapy, University Medical Center Freiburg, Freiburg, Germany; 4 Department of Medical Psychology, University Medical Center Hamburg Eppendorf, Hamburg, Germany; 5 Practice for Gastroenterology and Oncology, Schwetzingen, Germany

**Keywords:** Shared decision making (SDM), Preference matching, Physician training program, Breast and colon cancer, Oncology

## Abstract

The aims of this study are to assess patients’ preferred and perceived decision-making roles and preference matching in a sample of German breast and colon cancer patients and to investigate how a shared decision-making (SDM) intervention for oncologists influences patients’ preferred and perceived decision-making roles and the attainment of preference matches. This study is a post hoc analysis of a randomised controlled trial (RCT) on the effects of an SDM intervention. The SDM intervention was a 12-h SDM training program for physicians in combination with decision board use. For this study, we analysed a subgroup of 107 breast and colon cancer patients faced with serious treatment decisions who provided data on specific questionnaires with regard to their preferred and perceived decision-making roles (passive, SDM or active). Patients filled in questionnaires immediately following a decision-relevant consultation (t1) with their oncologist. Eleven of these patients’ 27 treating oncologists had received the SDM intervention within the RCT. A majority of cancer patients (60%) preferred SDM. A match between preferred and perceived decision-making roles was reached for 72% of patients. The patients treated by SDM-trained physicians perceived greater autonomy in their decision making (*p* < 0.05) with more patients perceiving SDM or an active role, but their preference matching was not influenced. A SDM intervention for oncologists boosted patient autonomy but did not improve preference matching. This highlights the already well-known reluctance of physicians to engage in explicit role clarification.

Trial Registration: German Clinical Trials Register DRKS00000539; Funding Source: German Cancer Aid.

## Introduction

Most cancer patients prefer collaborative roles with their oncologists in treatment decision making [9]. However, there is evidence that patients persistently experience less involvement in the decision-making process than they would like [3, 5, 17, 18, 22, 24, 31], and this mismatch has been linked to adverse patient outcomes such as anxiety, dissatisfaction, and decision regret [16, 18, 21, 24].

At least three approaches to medical decision making in which patients and physicians play different roles and that vary with regard to degree of patient autonomy have been described in the literature [7, 8, 12]. The shared decision-making (SDM) model, characterised by an intermediate degree patient autonomy, has been referred to as the “collaborative” model. Under the SDM model, both the patient and physician are actively involved in deciding on a plan of treatment: they exchange information, address expectations, concerns and fears, deliberate on treatment and role preferences, and finally arrive at a joint decision [7, 8, 12]. Another model is the paternalistic model, characterised by the lowest possible degree of patient autonomy. This model has been referred to as the “passive” or “doctor-directed” model because, under this approach, the physician alone decides, conveys his treatment decision to the patient, and thus takes sole responsibility for the treatment plan. The final model is the information model (or consumer model), which involves the highest possible degree of patient autonomy. Under this model, the patient is the sole independent and responsible decision maker, making decisions after receiving complete medical information from the physician. This model has been referred to as the “active” or “patient-directed” model.

Studies of cancer patients’ decision-making preferences generally report the highest preferences for SDM [5, 9, 16, 18, 24, 30], and the desire for this approach to decision making has increased over the last three decades [9]. Two authors have closely analysed the decision-making preferences and experiences of cancer patients in the German healthcare setting [15, 16, 33]. In contrast to international studies, the findings of Vogel et al. [33] suggest that paternalism is more widely accepted among German patients and that SDM appears to be rare in the initial treatment of breast cancer patients. Ernst et al. [15] surveyed 533 German cancer patients, finding varying preferences with respect to participation, depending on the issue at hand. Patients who reached a preference match and those who were over-involved reported the highest satisfaction levels.

Because international evidence strongly supports the benefits and popularity of SDM, physicians have been encouraged to involve patients in their treatment decisions. It is recommended that physicians assess patients’ preferences with respect to participation and match their consultation approach to these preferences [25, 28, 29]. However, it is extremely challenging for physicians to flexibly match their consultation styles to the decision-making preferences of individual patients. The percentage of patients who fulfil their desired role in decision making ranges from 34 to 80% [22, 25, 29]. Mismatches are most often found among patients who feel less involved than they wish to be. Therefore, several authors have called for communication training programs that teach physicians SDM and preference assessment skills [17, 25, 33]. A framework for teaching and learning SDM involving several steps has been proposed and should ideally be followed [13, 32]. One of these steps—referred to as role clarification—is to explicitly assess patients’ preferences with respect to their roles in the decision-making process. Physicians who have received SDM training should thus be able to better match their consultation style to the decision-making preferences of individual patients.

To date, the influence of an SDM training program on patients’ perceived decision-making roles and the achievement of preference matching has not been thoroughly examined. The International Breast Cancer Study Group recently assessed the effects of a 7-h physician communication training program that focuses on SDM in a large patient sample (*n* = 683) [3], finding a desire for more involvement in decision making among the training group than the control group, although the difference was not statistically significant.

In summary, several studies have underscored physicians’ shortcomings in involving cancer patients in medical decision making and in matching their consultation style to patients’ desired levels of autonomy, but benefits for patients can be expected under both approaches. The situation for cancer patients in Germany appears to be even more desperate. In light of these findings, we explored the following questions:

1. How are patients’ preferred and perceived decision-making roles distributed among a sample of German breast and colon cancer patients?
2. How many cancer patients attain a preference match?
3. How does an SDM training intervention for oncologists influence patients’ preferred and perceived decision-making roles and the attainment of a preference match?

## Methods

The participants were a subgroup of 86 physicians and their 160 patients with breast or colon cancer who participated in a prospective parallel-group cluster RCT and provided data on specific questionnaires relevant for the analysis reported in this paper. The study reported here is a post hoc analysis of the RCT. Detailed results of the RCT as well as recruitment methods and procedures are reported elsewhere [19] and will therefore only be summarised. In the RCT, patient-reported outcomes and observer-rated measures (OPTION) of an intervention group (IG) (SDM intervention for physicians) and a control group (CG) (treatment as usual) were compared. The main result of the RCT was that SDM-trained IG physicians showed higher competence than their non-trained counterparts in observer-rated SDM skills (Cohen’s *d* = 0.56; *p* < 0.05). Patients treated by trained physicians had lower anxiety and depression scores immediately after the consultation (*d* = −0.12 and −0.14, respectively; *p* < 0.10), and markedly lower anxiety and depression scores 3 months later (*d* = −0.94 and −0.67, *p* < 0.01). The ethics committees of the University of Heidelberg, Germany, and the University of Freiburg, Germany, approved the trial.

### Participants

#### Physicians

In total, 86 physicians participated in the RCT and were randomised to IG or CG. However, 53 physicians dropped out from the study due to organisational barriers like job rotations and time constraints before recruiting any patients. Six more physicians were excluded from the analysis because their patients did not provide valid data on the Control Preferences Scale (CPS) or the Patient Perception Scale (PPS). Thus, the analysis included 27 physicians, 11 of whom (40.7%) had been trained in SDM (IG). The physicians’ ages ranged from 26 to 58 years (*M* = 37.44, *SD* = 7.81), and their professional experience ranged from 6 months to 30 years (*M* = 9.72, *SD* = 7.16). Fifteen of the 27 physicians (55.6%) were male. Twenty-four physicians (88.9%) worked in an inpatient setting. The majority of physicians (*n* = 21, 77.8%) had no training in psychosomatics.

#### Patients

In total, 160 patients with breast or colon cancer faced with a serious treatment decision (i.e., decision for or against adjuvant chemotherapy in patients with colon cancer stage II with risk factors or decision for mastectomy or lumpectomy and radiation in early-stage breast cancer) participated in the RCT. The patients’ socio-demographic and clinical characteristics are summarised in Table 1. Fifty-three patients were eliminated from the analysis because they did not provide data on the CPS or PPS. Significantly more females than males did not complete the CPS (*χ* ^2^(1, *N* = 133) = 4.41, *p* = .04). There were no other significant differences with respect to socio-demographic characteristics between those who completed the CPS or PPS and were analysed and those who were eliminated from the study prior to the analysis. Of the 107 patients included in the analysis, 93 were inpatients (86.9%). Of these, 78.5% (*n* = 84) were women. The patients’ ages ranged from 25 to 88 years (*M* = 63.78; *SD* = 13.51). The majority of the patients had early-stage disease, and the most common diagnosis was breast cancer (54.2%).

**Table 1:** Socio-demographic and clinical characteristics of the patient sample (*N* = 107)

	Control group (*n* = 71), *n* (%)	Intervention group (*n* = 36), *n* (%)	Total (*N* = 107), *n* (%)
Sex			
Male	12 (16.9)	11 (30.6)	23 (21.5)
Female	50 (82.0)	25 (69.4)	84 (78.5)
Age (years)			
Mean (SD)	64.0 (12.4)	63.4 (15.6)	63.8 (13.5)
Family status			
Never married	7 (7.0)	2 (5.6)	7 (6.5)
Married	44 (62.0)	25 (69.4)	68 (63.6)
Divorced	8 (11.3)	3 (8.3)	11 (10.3)
Widowed	14 (19.7)	6 (16.7)	20 (18.7)
Formal education			
Below 12 years	61 (85.9)	33 (91.7)	94 (87.9)
12 years or more	9 (12.7)	3 (8.3)	12 (11.2)
Cancer type			
Breast	44 (62.0)	14 (38.9)	58 (54.2)
Colon	27 (38.0)	22 (61.1)	49 (45.8)
Cancer stage			
I	18 (25.4)	9 (25.0)	27 (25.2)
II	17 (23.9)	10 (27.8)	27 (25.2)
III	24 (33.8)	10 (27.8)	34 (31.8)
IV	8 (11.3)	4 (11.1)	12 (11.2)

Cells not adding up to column sums indicate missing values; valid relative frequencies are reported

### Procedure

After consenting to participate in the study, the physicians completed a questionnaire on their socio-demographic and work-related characteristics and were randomly allocated to the IG or CG. Physicians in the IG received 12 h of SDM training that included risk communication and the use of decision boards [19] before starting patient recruitment. The CG physicians immediately started patient recruitment. As an incentive to participate in the study, the CG physicians were offered SDM training after completion of the RCT. Each physician was asked to include eight patients in the study. The patients were informed about the study by their physician, received written study information, and signed informed consent forms. After the initial consultation, which was audio-taped by the physician, the patients were asked to directly complete a patient questionnaire (t1) and return it in a post-paid return envelope.

### Measures

#### Control Preferences Scale

The patients’ role preferences for involvement in the decision-making process were assessed using the Control Preferences Scale (CPS) [11]. The patients were asked to indicate how they would like their treatment decisions to be made. Response options included the following: (a) “I prefer to make the final treatment decision”; (b) “I prefer to make the final treatment decision after seriously considering my doctor’s opinion”; (c) “I prefer that my doctor and I share responsibility for deciding which treatment is best”; (d) “I prefer that my doctor makes the final treatment decision but seriously considers my opinion”; and (e) “I prefer to leave all treatment decisions to my doctor”. In the analyses, the responses were collapsed into three categories to reflect a paternalistic/passive approach (options d and e), an SDM/collaborative approach (option c) and an information model/active approach (options a and b) [11].

#### Patient Perception Scale

The patients’ role perceptions in the decision-making process were assessed using adaptations of the above noted items from the CPS in which patients indicated their perceptions of what actually occurred in the consultation [23]. In this Patient Perception Scale (PPS) they could again choose from five statements that described how the decision was made, e.g., “My doctor and I shared responsibility for deciding which treatment was best for me”. For the purposes of the analyses, the five categories were again collapsed into the three abovementioned categories (see also [10, 27]).

#### Preference Match

To describe how a patient’s perceived decision-making role (PPS) [23] accorded with his/her preferred decision-making role (CPS) [11], a new variable, indicating three levels of congruence, was created from the responses to the PPS and CPS: (1) the patient participated in the decision at a level that was less than he/she preferred (under-involved); (2) the patient’s experience was concordant with his/her preference (successful match); (3) the patient participated in the decision at a higher level that he/she preferred (over-involved).

### Data Analysis

Descriptive statistics (frequencies and percentages) were used to describe socio-demographic, disease-related (patients) and work-related (physicians) data, role preferences, and perceived roles. Using *χ* ^2^ tests, the proportions of patients were compared with respect to their answers to questions regarding decision-making roles. Differences between the IG and CG with respect to patients’ preferred and perceived decision-making roles and preference matching were also assessed using *χ* ^2^ tests.

## Results

### Preferred and Perceived Decision-Making Roles

**Fig. 1:**
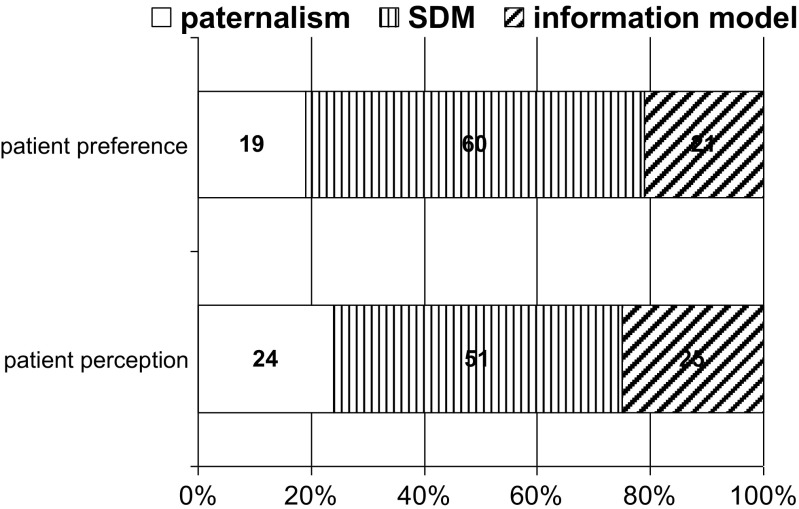
Distribution of cancer patients’ decision-making preferences and perceptions: patients’ preferred decision-making roles (*n* = 107) and patients’ perceived decision-making roles (*n* = 97) in percentages

Figure 1 presents the patients’ decision-making preferences and experiences. We found that 59.8% (*n* = 64) of all breast and colon cancer patients preferred to share treatment decisions with their physicians. Additionally, 20.6% (*n* = 22) of patients wished to make the treatment decision on their own, and 19.6% (*n* = 21) of patients preferred that the physician make the treatment decision. More than half of the patients (51.5%, *n* = 50) reported that the actual decision making was shared (SDM), 24.7% (*n* = 24) reported an active decision-making experience (information model), and 23.7% (*n* = 23) reported that the decision-making was physician-directed (paternalism).

### Match of Preferred and Perceived Decision-Making Roles

Ninety-six patients provided data on both the CPS and PPS (see Table 2). Of these, 71.9% (*n* = 69) reported a match between their preferred and perceived decision-making approaches (Table 2 diagonal), 13.5% (*n* = 13) felt that they had been less involved than they preferred, and 14.6% (*n* = 14) felt more involved than they preferred.

**Table 2:** Patients’ preferred and perceived decision-making roles and their match or mismatch

	Perceived decision-making role (post-consultation)							
	Paternalistic model		SDM		Information model		Totals	
Preferred decision-making role (post-consultation)	*n*	%	*n*	%	*n*	%	*N*	%
Paternalistic model	**13**	**13.5**	3	3.1	4	4.2	20	20.8
SDM	8	8.3	**43**	**44.8**	7	7.3	58	60.4
Information model	2	2.1	3	3.1	**13**	**13.5**	18	18.8
Totals	23	24.0	49	51.0	24	25.0	96	100.0

More involvement than preferred above diagonal: 14 patients, 14.6%. Less involvement than preferred below diagonal: 13 patients, 13.5%. Bold indicates preference match. Missing *n* = 11 (10.3%)

*SDM* shared decision-making

### Influence of a Shared Decision-Making Training Intervention for Physicians on Patients’ Preferred and Perceived Decision-Making Roles and on Preference Matching

**Fig. 2:**
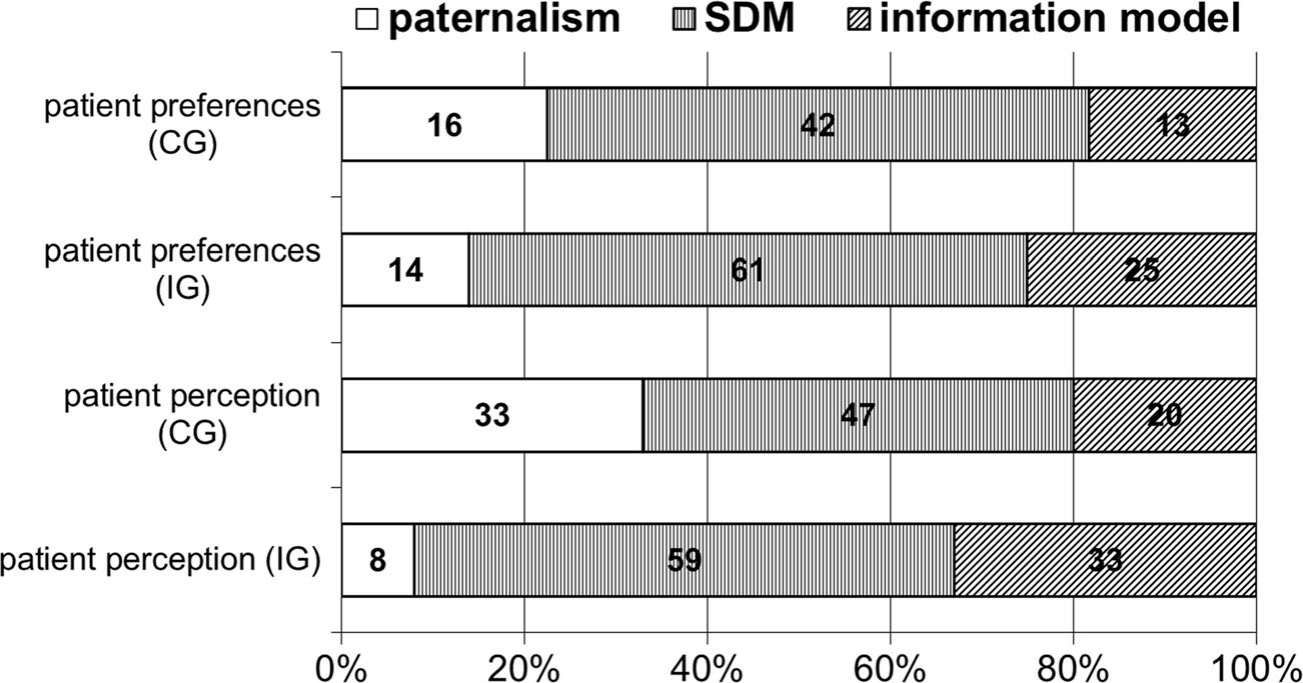
Influence of SDM intervention on patients’ decision-making preferences and perceptions: patients’ preferred decision-making roles (*n* = 107) and patients’ perceived decision-making roles (*n* = 97) in the control group (CG) and the intervention group (IG) in percentages

Preferred and perceived decision-making roles of IG and CG patients are presented in Fig. 2. Chi-squared analyses show significant group differences in the PPS (*χ* ^2^(2, *N* = 97) = 7.93, *p* = 0.019), with CG patients more likely than IG patients to experience paternalism (paternalism IG: *n* = 3 (8.3%) vs. CG: *n* = 20 (32.8%) and with IG patients more likely than CG patients to experience SDM (IG: *n* = 21 (58.3%) vs. CG: *n* = 29 (47.5%)) or information model (IG: *n* = 12 (33.3%) vs. CG: *n* = 12 (19.7%). Additionally, 72% (*n* = 44) of CG patients and 71% (*n* = 25) of IG patients achieved their preferred level of involvement. There were no significant group differences neither on the CPS (*χ* ^2^(2, *N* = 107) = 1.45, *p* = 0.485) nor on preference matching (*χ* ^2^(2, *N* = 96) = 0.43, *p* = 0.806).

## Discussion

In this study, we assessed a sample of 107 German breast and colon cancer patients faced with serious treatment decisions with regard to preferred and perceived roles in medical decision making and successful preference matching. In particular, we assessed the influence of an SDM intervention on these three variables.

The study’s first main finding is that the desire for SDM was predominant among surveyed German cancer patients, with 60% of patients opting for a collaborative SDM approach with their physician independent of the group condition (IG vs. CG). These findings indicate that involvement in treatment decision making is a development welcomed by cancer patients in Germany, a conclusion that contrasts with the findings of Vogel et al. [33], who reported the predominance of passive role preferences (40%) in a sample of newly diagnosed German breast cancer patients. While 29% of patients in their sample preferred SDM, only 13% reported having experienced SDM. Vogel et al. [33] suspected that cultural differences and characteristics of German health services explained their findings, as these findings contrast with those of other international studies [5, 9, 22, 23, 26], which have reported higher involvement preferences. Another assessment of German cancer patients [15] produced findings closer to international samples. However, Vogel et al. [33] concluded that SDM is rare in initial treatments of breast cancer patients in Germany. In our study, even in the control group, 42% of patients felt after consultation that SDM has been achieved. This finding casts a more favourable light on the practice and on the ability of German oncologists to involve their patients in treatment decision making. One possible reason for the higher patient involvement documented in our sample may be the physicians’ awareness of patient involvement as the focus of our study, which may have resulted in a high motivation to comply with this aim. Another reason may relate to a time trend reported in a review [9] showing that the number of patients preferring a collaborative SDM approach has increased internationally over the past three decades. This time trend may follow a different dynamic in Germany, where it may have started later and increased at a fast pace, thanks to a decade of substantial advances in German health policy that have fostered increased socio-political acceptance of SDM [20].

The study’s second main finding is that matching of patients’ preferred and perceived decision-making styles was achieved for 72% of the cancer patients in our sample. Patients whose preferences were not met reported either being more involved (15%) or less involved (14%) than they wished to be. This rate of preference matching is at the upper end of the spectrum found in most studies that have investigated this issue; previously reported percentages have ranged between 34 and 80% [25]. For the two German samples, 44% [15] and 63% [33] of patients achieved a preference match. Our findings might indicate that the physician sample in our study was rather sensitive and skilled in matching their decision-making style to their patients’ desired levels of participation.

The study’s third main finding is that the SDM intervention was successful in boosting patient autonomy because it significantly raised the extent of involvement patients experienced in their consultations. Altogether, 92% of IG patients perceived collaborative or active decision making, and only 8% experienced paternalism. In the CG, 67% of patients experienced some degree of involvement (collaborative or active), and 33% reported paternalism. Thus, the “baseline” level of involvement in the CG is already satisfactory. We assume that study participation per se may have increased physicians’ awareness of the importance of patient involvement and may have prompted them to facilitate patient involvement as best they could. However, as we hoped to demonstrate, physicians enrolled in SDM training showed higher involvement skills than their counterparts not enrolled in SDM, skills that—because the design was randomised controlled—they must have acquired during the training. In line with this result, findings discussed in the RCT publication of the trial [19] show that SDM-trained physicians demonstrated better observer-rated SDM skills than the control physicians when audio-tapes of the consultations were analysed using the OPTION instrument. The International Breast Cancer Study Group recently assessed the effects of a 7-h communication training program for physicians, with a focus on SDM [3]. They found a trend towards greater patient involvement in decision making in the training group compared with the CG but no significant differences. They concluded that their 7-h SDM training program may not have been sufficiently intensive to produce patient-related outcomes, as a dose-response effect is known to generally characterise communication skills training. However, our slightly more intensive SDM training program, in combination with the use of decision boards, led to an increase in patient autonomy, which suggests that a satisfactory training dose for this purpose may be 12 h or more.

Contrary to expectations, we detected no influence of the SDM intervention on preference matching. Irrespective of whether an SDM intervention was administered, a preference match was attained for approximately 72% of patients (IG 71%, CG 72%). Ideally, preference matching should have been more common in the IG than in the CG because one of the six steps of the SDM model [13] taught during training is role clarification. However, CG and IG patients differed with respect to unmet expectations. Specifically, CG patients might have experienced more unwanted paternalism, while the IG patients might have been pushed into an overly active role. Our findings support the power of SDM training to strengthen patient autonomy, but they also demonstrate a need to intensify the module on eliciting patients’ preferred decision-making roles and adhering to them. Indeed, in the SDM training program, our trainers found that the physicians felt uncomfortable in role playing when asked to engage in explicit role clarification. The reluctance of physicians to engage in explicit role clarification has been previously observed [14]. Analyses of audio-taped consultations using the observer-based OPTION scale found shortcomings among nearly all physicians with respect to the SDM step of role clarification (unpublished data). As a consequence, we will revise the role clarification module of the SDM training program for future use. However, we do not know if an unmatched preference and perceived decision-making style lead to a negative evaluation of the consultation by the patients. Especially for patients who prefer SDM and perceive an over-involvement (information model), it might be considered that a consultation in a SDM style supports the autonomy of the patient in a way that the patient is comfortably able to make a treatment decision without a recommendation of the physician.

The study’s findings must be seen in light of some limitations. Despite a sufficient number of participating physicians, we failed to reach the targeted sample size for patients (eight for each physician) in the RCT. We suspect that, among other reasons, this relatively small patient pool relates to the demanding study procedures for the physicians and their reluctance to audio-tape presumably imperfect consultations (also see [19]). The small sample size of patients may have hindered detection of small effects due to the underpowered nature of the study. However, and because of this, the detected effects can be considered all the more robust. Some of the effects found in the study, such as the rather high involvement rates and the high rates of preference matching attained, may result from self-selection bias, with physicians who are open to the SDM concept more likely to participate in the study [4]. Although study participation was usually imposed in a top-down manner by the chief consultants of the whole physician team at the cancer centre, a consultant’s openness to SDM may rub off on the whole team. Another weakness of the study is the uneven allocation of patients to the IC and CG treatments, with more than half allocated to the CG, impeding subgroup analyses. This most likely occurred because CG physicians could start patient recruitment immediately after study inclusion and did not have to undergo SDM training first. Additionally, Brown et al. [5] have shown that role preferences may differ when they are assessed before and after the consultation. Post-consultation preferences in their study were more likely to accord with patients’ perceived decision-making approach. In our study, we only assessed post-consultation role preferences of patients and may therefore have missed the share of patients who altered their preferences post-consultation.

Future studies that assess cancer patients’ preferences and perceptions of involvement should keep patient recruitment as uncomplicated as possible for physicians to prevent high physician dropout rates.

## Conclusion

The desire for involvement in treatment decision-making was high in the sample of German breast and colon cancer patients surveyed in this study, and 72% of the sample attained their preferred role in decision making. The 12-h SDM training program, in combination with use of decision boards, boosted patient autonomy but did not lead to greater consideration of patients’ individually preferred decision-making style. This highlights the already well-known reluctance of physicians to engage in explicit role clarification. Consideration of patients’ preferred decision-making style is of high importance; however, when in doubt, physicians should offer SDM as the safest alternative.
